# A one-step procedure for immobilising the thermostable carbonic anhydrase (SspCA) on the surface membrane of *Escherichia coli*

**DOI:** 10.1080/14756366.2017.1355794

**Published:** 2017-08-09

**Authors:** Sonia Del Prete, Rosa Perfetto, Mosè Rossi, Fatmah A. S. Alasmary, Sameh M. Osman, Zeid AlOthman, Claudiu T. Supuran, Clemente Capasso

**Affiliations:** a Dipartimento di Scienze Bio-Agroalimentari, CNR-Istituto di Bioscienze e Biorisorse, CNR, Napoli, Italy;; b Dipartimento Neurofarba, Sezione di Scienze Farmaceutiche, and Laboratorio di Chimica Bioinorganica, Polo Scientifico, Università degli Studi di Firenze, Florence, Italy;; c Department of Chemistry, College of Science, King Saud University, Riyadh, Saudi Arabia

**Keywords:** Carbonic anhydrase, thermostable enzyme, ice nucleation protein, hydratase activity, protonography, outer membrane

## Abstract

The carbonic anhydrase superfamily (CA, EC 4.2.1.1) of metalloenzymes is present in all three domains of life (Eubacteria, Archaea, and Eukarya), being an interesting example of convergent/divergent evolution, with its seven families (α-, β-, γ-, δ-, ζ-, η-, and θ-CAs) described so far. CAs catalyse the simple, but physiologically crucial reaction of carbon dioxide hydration to bicarbonate and protons. Recently, our groups characterised the α-CA from the thermophilic bacterium, *Sulfurihydrogenibium yellowstonense* finding a very high catalytic activity for the CO_2_ hydration reaction (*k*_cat_ = 9.35 × 10^5^ s^−1^ and *k*_cat_/*K*_m_ = 1.1 × 10^8^ M^−1 ^s^−1^) which was maintained after heating the enzyme at 80 °C for 3 h. This highly thermostable SspCA was covalently immobilised within polyurethane foam and onto the surface of magnetic Fe_3_O_4_ nanoparticles. Here, we describe a one-step procedure for immobilising the thermostable SspCA directly on the surface membrane of *Escherichia coli*, using the INPN domain of *Pseudomonas syringae*. This strategy has clear advantages with respect to other methods, which require as the first step the production and the purification of the biocatalyst, and as the second step the immobilisation of the enzyme onto a specific support. Our results demonstrate that thermostable SspCA fused to the INPN domain of *P. syringae* ice nucleation protein (INP) was correctly expressed on the outer membrane of engineered *E. coli* cells, affording for an easy approach to design biotechnological applications for this highly effective thermostable catalyst.

## Introduction

Different expression systems have been used to obtain recombinant proteins, such as *Escherichia coli* ^1^ ^,^ ^2^, *Saccharomyces cerevisiae* ^3^, *Pichia pastoris* ^4^, baculovirus/insect cells^1^ ^,^ ^2^ ^,^ ^5^, mammalian cell lines^6^, and cell-free *in vitro* protein production systems^7^. These methods have advantages and disadvantages depending on the type of protein to be expressed^8^. For example, the *E. coli* heterologous expression of glycosylated macromolecules or high disulphide content proteins has the disadvantage that often the recombinant proteins are produced as insoluble or non-functional molecules^8^. In this case, it is preferable to switch to a different expression system, such as *Pichia pastoris*, baculovirus/insect cell system or baculovirus variants that promote greater protein secretion as well as post-translational modifications typical of eukaryotic cells^5^. However, these expression systems are very expensive with respect to the *E. coli* system, which is still the preferred host for the heterologous expression of recombinant proteins due to cost considerations, speed, ease of use and genetic manipulation^1^. For this reason, in commerce, it is possible to find *E. coli* strains capable of overcoming the problems of inefficient disulphide bond formation or engineered *E. coli* cells to perform protein N-glycosylation, even though the efficiency is generally very low^9^. Consequently, many attempts were made to efficiently produce a recombinant protein in *E. coli*, which remains the most common expression host^1^ ^,^ ^9^. In this context, a system has been developed for anchoring heterologous proteins or polypeptides on the outer surface membrane of *E. coli* using the ice nucleation protein (INP) of the Gram-negative bacterium, *Pseudomonas syringae* ^10^. INP is an outer membrane protein capable of imparting ice crystal formation on the supercooled water, with a deduced molecular weight of 118 kDa^10^. It has been demonstrated that the N-terminal region (INPN, about 18 amino acid residues) of this protein seems to interact with the phospholipid moiety of the bacterial outer membrane. Its central domain (of about 96 amino acid residues) is composed of repeats given by an 8-, 16-, and 48-residue periodicity that acts as the template for ice crystal formation, whereas the C-terminal region (INPC, about five amino acid residues) is highly hydrophilic and exposed to the outermost cell surface^10^. The heterologous expression of proteins on the bacterial surface requires some essential requirements, such as (i) a signal peptide-type sequence to make possible the translocation of the neo-synthetised protein through the cytoplasmic membrane, (ii) a guiding motif to reach to the cell surface, and (iii) an anchoring motif to attach the protein to the bacterial surface^10^. From previous studies, Fan et al. have demonstrated that for a high expression level of the host protein on the bacterial surface it is sufficient the guiding motif and the anchoring motif of the ice nucleation protein (INP). These two motifs include the N-amino terminal region and the first two subunits of the central domain of *P. syringae* INP protein^11^. They form the INPN domain necessary to display the protein on the cell surface^11^. In this way, the INPN domain is located in the outer membrane and the recombinant protein is stably exposed on the external side of the bacterial outer membrane^11^. By using the INPN domain of *P. syringae,* we expressed on the bacterial surface of *E. coli*, the thermostable carbonic anhydrase, SspCA, already characterised by our groups and^12–23^ identified in the genome of the species YO3AOP1 of *Sulfurihydrogenibium*, isolated in the Yellowstone National Park, USA^24^. Carbonic anhydrase superfamily (CAs, EC 4.2.1.1) of metalloenzymes has been found in all the three domains of life (Eubacteria, Archaea, and Eukarya)^25–42^ and are an example of convergent/divergent evolution phenomena, with seven known families to date, the α-, β-, γ-, δ-, ζ-, η-, and θ-CAs^43–45^. CAs catalyse the simple but physiologically crucial reaction of carbon dioxide hydration to bicarbonate and protons: CO_2_ + H_2_O ⇄ HCO_3_^−^ + H^+46–48^ with a *k*_cat_ ranging from 10^4^ to 10^6^ s^−1^ making faster the naturally reversible but slow CO_2_ hydration reaction, due to the slow rate of carbonation reaction (10^−1 ^s^−1^)^16^ ^,^ ^40–42^ ^,^ ^49–71^. In this study, SspCA was selected as a model protein because recently, thermostable or non-thermostable CAs were covalently immobilised on different supports, in order to be used in the biomimetic CO_2_ capture processes or for other biotechnological applications. Thus, using the INPN domain strategy, SspCA was produced and directly immobilised in a one-step procedure on the bacterial surface (of *E. coli*) during its overexpression. This is clearly an advantage with respect to methods, which require as the first step the production and the purification of the biocatalyst, and, as the second step, the immobilisation of the enzyme on a specific support^23^ ^,^ ^72^. Therefore, the one-step procedure here reported drastically reduces the cost of the enzyme purification to be used for the covalent immobilisation and the cost of the support necessary for the biocatalyst immobilisation steps. Our results demonstrated that SspCA was efficiently overexpressed and active on the bacterial surface of *E. coli*. Moreover, this strategy could be used to further improve the whole cell capture procedures of carbon dioxide with surface-expressed SspCA.

## Materials and methods

### Chimeric gene and plasmid preparation

To construct the INPN domain-SspCA surface-anchoring vector, it has been considered the nucleotide sequence encoding for the INPN domain with the first two subunits, identified in the genome of *P. syringae*, and the nucleotide sequence of the thermostable α-CA, SspCA, identified in the genome of the thermophilic bacteria *S. yellowstonense*. In details, we designed a chimeric gene composed of the following fragments: (a) the INPN domain, a nucleotide fragment of 612 bp encoding for the *N*-terminal of the INP (ice nucleation protein) and containing the two first subunits of INP; (b) the nucleotide spacer of 15 bp encoding for the following amino acid residues EAYGS; and (c) the SspCA gene, a nucleotide sequence of 678 bp encoding for the thermostable α-CA, SspCA, lacking of the peptide signal (the first 20 amino acids of the peptide sequence). Moreover, the complete chimeric gene contained an EcoR*I* and Hind*III* restriction site at the 5′ and 3′ end of gene, respectively. The chimeric gene was indicated with the acronym INPN-SspCA and inserted in the plasmid pMKA. The resulting plasmid was amplified into *E. coli* DH5 α cells. As expression vector to obtain the surface display of the fusion chimeric gene, it has been chosen the pET-22 b(+) vector (Novagen, Madison, WI) because it carries an N-terminal pelB, the signal sequence for the periplasmic translocation of the protein, and a C-terminal His-Tag sequence for protein purification and identification. Thus, INPN-SspCA was digested with EcoR*I* and Hind*III*, and then ligated with T4 DNA ligase into the same sites of pET-22 b(+) expression vector to give the expression vector pET-22 b/INPN-SspCA. In order to confirm the integrity of the INPN-SspCA gene in the pET-22 b(+) vector and that no errors had taken place at the ligation sites, the vector containing the fragment was bi-directionally sequenced. The final construct contained a nucleotide sequence expressing a chimeric protein formed by the pelB signal peptide of 21 amino acid residues, the INPN domain of 204 amino acid residues, the spacer of five amino acid residues, the thermostable SspCA of 226 amino acid residues, and a tail of six histidines at the C-terminus of the SspCA sequence.

### INPN-SspCA cell surface expression

Competent *E. coli* BL21 (DE3) cells were transformed with pET-22 b/INPN-SspCA, grown at 37 °C, and when cells had grown to an OD_600_ of 0.6–0.7, the protein surface expression was induced with 1.0 mM isopropyl-thio-b-d-galactoside (IPTG) and 0.5 mM ZnSO_4_. After additional growth for 6 h, the cells were harvested by centrifugation and washed three times with PBS. It has been recovered about 4 g of whole cells. Aliquots of cells were resuspended in 25 mM Tris/HCl and used to determine the enzyme activity on the whole cells or to prepare the outer membrane fraction.

### Outer and inner membrane fractionation

To perform the separation of the outer and the inner membrane of the *E. coli* strain, 2 g of harvested bacterial cells were resuspended in a 40 ml of 25 mM Tris/HCl buffer, pH 8.0 and disrupted by sonication (10 s, for 10 cycles) on the ice. Cell extract was centrifuged at 40,000 rpm for 1 h using an ultracentrifuge. After the ultracentrifugation, the supernatant containing the soluble cytoplasmic fraction was discarded, while total membrane fraction was recovered in the pellet. For obtaining the outer membrane fraction, the pellet was resuspended in 40 ml of phosphate-buffered saline (PBS) containing 0.01 mM MgCl_2_ and 2% Triton X-100 and incubated at room temperature for 30 min to solubilise the inner membrane. The outer membrane fraction was then repelleted by ultracentrifugation at 40,000 rpm. The outer membrane pellet containing the membrane surface SspCA was used for further experiments, such as enzyme activity, SDS page, Western blot, and thermostability.

### Carbonic anhydrase assay of the free and membrane-bound enzyme

CA activity assay was a modification of the procedure described by Capasso et al.^14^. Briefly, the assay was performed at 0 °C using CO_2_ as substrate following the pH variation due to the catalysed conversion of CO_2_ to bicarbonate. Bromothymol blue was used as the indicator of pH variation. The production of hydrogen ions during the CO_2_ hydration reaction lowers the pH of the solution until the colour transition point of the dye is reached. The time required for the colour change is inversely related to the quantity of CA present in the sample. Wilbur–Anderson units were calculated according to the following definition: One Wilbur–Anderson unit (WAU) of activity is defined as (*T*_0_ − *T*)/*T*, where *T*_0_ (uncatalysed reaction) and *T* (catalysed reaction) are recorded as the time (in seconds) required for the pH to drop from 8.3 to the transition point of the dye in a control buffer and in the presence of enzyme, respectively. Assay of the free enzyme was carried out using about 100 ng of previously purified free SspCA^16^ ^,^ ^17^ ^,^ ^23^ ^,^ ^62^, while for the membrane-bound enzyme was used an amount of whole cells or outer membrane ranging from 1 to 5 mg.

### Sodium dodecyl sulphate (SDS)-polyacrylamide gel electrophoresis (PAGE)

SDS-PAGE was performed as described by Laemmli using 12% gels^73^. Samples were dissolved in buffer with 5% β-mercaptoethanol. The gel was stained with Coomassie blue. Protein concentration was determined by Bio-Rad assay kit (Bio-Rad, Hercules, CA).

### Protonography

Wells of 12% SDS-PAGE were loaded with whole cells, outer membrane, inner membrane, cytoplasmic fraction coming from the whole cell lysates, and purified free SspCA mixed with loading buffer without 2-mercaptoethanol and without boiling the samples, in order to solubilise cells and avoid protein denaturation. The gel was run at 180 V until the dye front ran off the gel. Following the electrophoresis, the 12% SDS-PAGE was subject to protonography to detect the INPN-SspCA hydratase activity on the gel as described by Capasso et al.^58^ ^,^ ^69^ ^,^ ^74^.

### His-Tag Western blotting

Protein and solubilised outer and inner membranes were subject to a 12% (w/v) SDS-PAGE, followed by electrophoretic transfer to a PVDF membrane with transfer buffer (25 mM Tris, 192 mM glycine, 20% methanol) by using Trans-Plot SD Cell (Bio-Rad, Hercules, CA). His-Tag Western blot was carried out using the Pierce Fast Western Blot Kit (Thermo Scientific, Waltham, MA). Blotted membrane has been placed in the wash blot solution Fast Western 1× Wash Buffer to remove transfer buffer. Primary Antibody Working Dilution was added to the blot and incubated for 30 min at room temperature (RT) with shaking. After, the blot was removed from the primary antibody solution and incubated for 10 min with the Fast Western Optimised HRP Reagent Working Dilution. Subsequently, the membrane was washed two times in about 20 ml of Fast Western 1× Wash Buffer. Finally, the membrane was incubated with the detection reagent working solution and incubated for 3 min at room temperature and then developed with X-ray film.

### Temperature studies on the free SspCA, whole cells surface, and outer membrane displaying SspCA

#### Effect of temperature

To compare the stability of the free SspCA and cell or outer membrane surface SspCA at different temperatures, free SspCA at the concentration of 1 mg/ml in 10 mM Tris/HCl, pH 8.3, cells displaying the SspCA (2 g/20 ml), or outer membrane SspCA (2 g/20 ml) were incubated at 25, 50, and 70 °C for different times (5, 7, 9, and 15 h). Free or cell/membrane bound enzymes aliquots were withdrawn at appropriate times and the residual activity was measured using CO_2_ as a substrate. All data have been analysed by means of GraphPad Prism 5.0 software (GraphPad Software, San Diego, CA). Curves were obtained by the mean of three independent determinations.

#### Long-term stability

Free SspCA and cell or outer membrane surface SspCA were examined for long-term stability (1, 2, 3, 4, 6, 8, and 10 d) at 25 °C by assaying their hydratase residual activities using CO_2_ as a substrate. Free or cell/membrane bound enzymes aliquots were withdrawn at appropriate times for the measurements of the long-term enzyme stability. All the buffers used for the long-term stability were sterilised by using a sterile 0.22 μm filter, while samples containing free or cell/membrane bound enzymes were treated with a diluted solution of sodium azide to avoid contamination. Aliquots of free SspCA and cell or outer membrane surface SspCA were withdrawn at different times and all data were obtained by the mean of three independent determinations.

### Free SspCA preparation

The recombinant free SspCA was prepared as described previously by Capasso et al.^14^.

## Results and discussion

### Expression and immobilisation of the membrane surface SspCA

**Figure 1.**
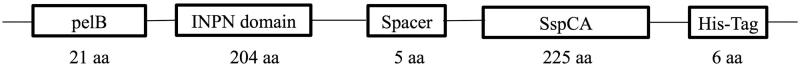
Schematic representation of the gene encoding the chimeric membrane bound SspCA. Legend: pelB, the signal sequence for the periplasmic translocation of the protein (21 amino acid residues); INPN domain (204 amino acid residues); Spacer (five amino acid residues); SspCA: the thermostable CA (226 amino acid residues); His-Tag: histidines at the C-terminus (6 amino acid residues).

**Figure 2.**
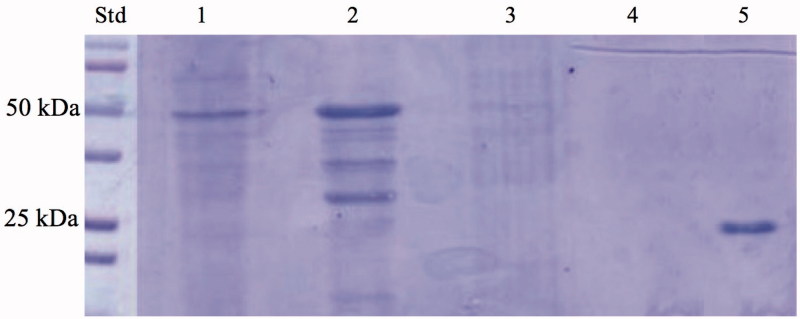
SDS-PAGE of the whole cells, outer membrane, inner membrane, cytoplasmic fraction coming from the whole cell lysates, and purified free SspCA. Legend: lane STD, molecular markers, M.W. starting from the top: 100 kDa, 75 kDa, 50 kDa, 37 kDa, 25 kDa, 20 kDa; lane 1, whole cells; lane 2, outer membrane; lane 3, inner membrane; lane 4, cytoplasmic fraction coming from the whole cell lysate; lane 5, purified SspCA. Lanes 1 and 2 showed a band at about 50 kDa corresponded to the INPN-SspCA, while lane 5 showed a band at about 25 kDa corresponded to the free SspCA. The band at a molecular weight of 50 kDa represented the overexpression of the chimeric SspCA, which was confirmed by the protonography and Western blot.

Recently, our groups reported the discovery and characterisation of an α-CAs from thermophilic bacteria belonging to the genus *Sulfurihydrogenibium*, living in hot springs all over the world, at temperatures of up to 110 °C^14^ ^,^ ^15^ ^,^ ^18^. The α-CA (SspCA) identified in the *S. yellowstonense* species had a high catalytic activity for the CO_2_ hydration reaction, with *k*_cat_ of 9.35 × 10^5^ s^−1^, *K*_m_ of 8.4 mM, and *k*_cat_/*K*_m_ of 1.1 × 10^8^ M^−1 ^s^−1^ (at 20 °C and pH of 7.5)^21^. Moreover, it retained its high catalytic activity (for the CO_2_ hydration reaction) even after being heated at 80 °C for several hours^13–15^ ^,^ ^17^ ^,^ ^21^. The molecular weight estimated by SDS-PAGE or calculated on the basis of the amino acid sequence translated from the gene was 26.0 kDA^21^. In 2014, we reported a three-phase trickle-bed reactor containing the highly thermostable SspCA covalently immobilised within polyurethane (PU) foam^72^. In 2017, we described the heterologous expression of the recombinant SspCA carried out using high-density fermentation of *E. coli* cultures, which was covalently immobilised onto the surface of magnetic Fe_3_O_4_ nanoparticles (MNP) by using the carbodiimide activation reaction. In the present manuscript, we describe a one-step procedure for immobilising the thermostable SspCA on the surface membrane of *E. coli*. Adding IPTG to the growing culture, the anchoring SspCA was overexpressed and directly immobilised on the outer membrane of *E. coli*. The chimeric polypeptide had a molecular weight of 50 kDa and it was a fusion of the following amino acid sequences: the pelB signal sequence for the periplasmic translocation (21 amino acid residues), the anchoring membrane INPN domain (204 amino acid residues), the spacer (5 amino acid residues), the thermostable α-CA, SspCA (226 amino acid residues), and a tail of six histidines at the C-terminus of the SspCA sequence (Figure 1). The SDS-PAGE carried out on the whole cells, on the fractioned outer and inner membrane, and on the cytoplasmic fraction confirmed the surface expression of SspCA (Figure 2). In fact, a band of 50 kDa and corresponding to the chimeric SspCA was only identified in the lanes corresponding to the whole cells and the outer membrane, while it was missed in the lanes of the cytoplasmic fraction and the inner membrane (Figure 2).

### Protonography and Western blot

**Figure 3.**
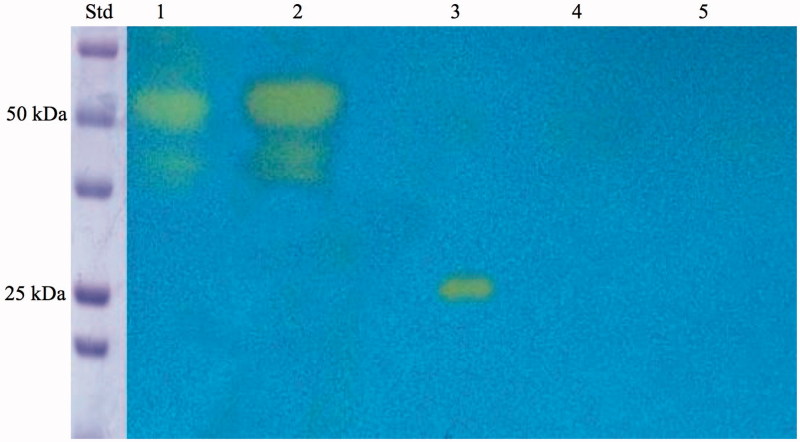
Protonography of the whole cells, outer membrane, inner membrane, cytoplasmic fraction coming from the whole cell lysates, and purified free SspCA. Legend: Lane STD, molecular markers, M.W. starting from the top: 75 kDa, 50 kDa, 37 kDa, 25 kDa, 20 kDa; lane 1, whole cells; lane 2, outer membrane; lane 3, purified SspCA; lane 4, inner membrane; lane 5, cytoplasmic fraction coming from the whole cell lysate. In lanes 1 and 2, the intense yellow band at the molecular weight of about 50 kDa represented the overexpression of the chimeric membrane-bound SspCA. The presence of the yellow bands below the intense band is due to the overloading of the sample on the gel. The yellow band of the lane 3 corresponds to the hydratase activity of the free SspCA. The lanes corresponding to the inner membrane and the cytoplasmic fraction did not evidence the chimeric membrane-bound SspCA.

**Figure 4.**
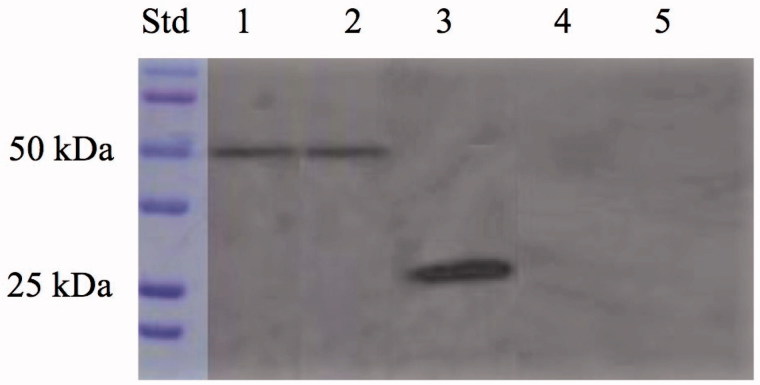
Western blot performed on the whole cells, outer membrane, purified free SspCA, inner membrane, and cytoplasmic fraction coming from the whole cell lysates. Legend: Lane Std, molecular markers, M.W. starting from the top: 100 kDa, 75 kDa, 50 kDa, 37 kDa, 25 kDa, and 20 kDa; lane 1, whole cells; lane 2, outer membrane; lane 3, purified SspCA; lane 4, inner membrane; lane 5, cytoplasmic fraction coming from the whole cell lysates. Lanes 1 and 2 showed a band at about 50 kDa corresponded to the INPN-SspCA, while lane 3 showed a band at about 25 kDa corresponded to the free SspCA. Both bands were identified using the anti-His-Tag antibody. Lanes 4 (inner membrane) and 5 (cytoplasmic fraction) did not evidence the presence of the chimeric SspCA.

The expression of the anchoring SspCA on the cell surface was confirmed by two techniques, which can be considered specific for the identification for the fusion SspCA. These biochemical techniques are the protonography and the Western blot. The protonography technique is based on the monitoring of pH variation in the gel (protonogram) due to the carbonic anhydrase catalysed the conversion of CO_2_ to bicarbonate and protons^25^ ^,^ ^33^ ^,^ ^40^ ^,^ ^69^ ^,^ ^74^ ^,^ ^75^. As expected, the production of hydrogen ions during the CO_2_ hydration reaction due to the hydratase activity of SspCA fused to the anchoring motif (INPN domain) determined the development of a yellow band only in the lanes containing the whole cells and the outer membrane fraction (Figure 3). Thus, the yellow band corresponded to the 50 kDa position on the gel confirmed that the surface membrane SspCA was anchored to the membrane of the whole cells and, precisely, to the outer membrane fractioned by ultracentrifugation (Figure 3). Western blot analysis also supported these results (Figure 4). The anchoring surface SspCA was prepared with a tail of six histidines at the C-terminus, thus using an anti-His-Tag antibody, it was found a clear expression of the fusion protein in the whole cells and outer membrane fraction at the molecular weight of 50 kDa, the molecular mass of the chimeric polypeptide chain (Figure 4). These demonstrated that the SspCA was efficiently expressed using an INP-based cell surface display system.

### SspCA activity onto cell surface

To verify the activity of the expressed SspCA on the bacterial cell surface, the hydratase activity of the whole cells and the outer membrane fraction was also determined in solution using a modification of the procedure described by Capasso et al.^14^ (see Material and methods section). Measuring the hydratase activity in solution using CO_2_ as a substrate, we found that 0.5 mg of whole cells or outer membrane fraction showed a hydratase activity corresponding to that obtained using 100 ng of the unbound enzyme. This result was similar to that obtained by our groups when the SspCA was covalently immobilised onto magnetic nanoparticles^23^. Probably, as happened for the magnetic nanoparticles, the enzyme immobilisation through the INPN membrane anchoring system determined a reduction of the three-dimensional conformational changes of the immobilised enzyme causing a reduction of the enzyme activity. Thus, it is necessary to use an amount of cells of about 5000 times more respect to the free enzyme, but the advantage of this expression system is that it is possible to obtain easily grams of bacterial cells having a surface localised SspCA activity. Moreover, as described below, the membrane immobilised SspCA had a higher stability both at room and high temperatures respect to the free enzyme.

### Effect of temperature on the cell surface SspCA

**Figure 5.**
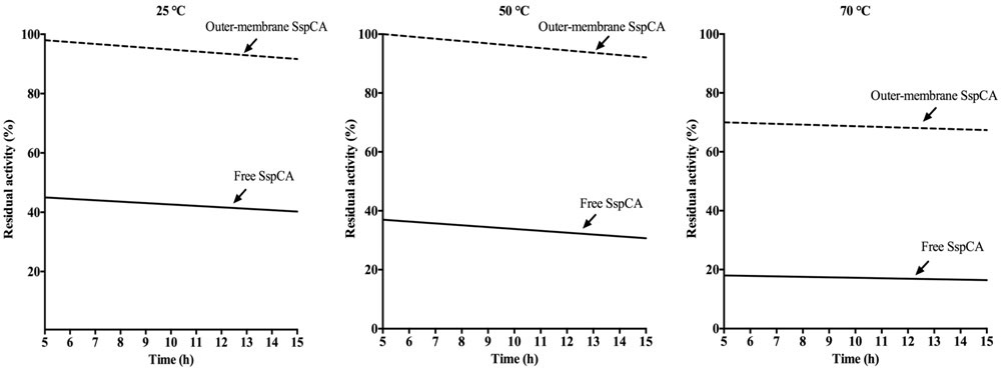
Temperature stability of the free SspCA and membrane-bound SspCA carried out at 25, 50 and 70 °C. Continuous line: free SspCA; dashed line: membrane-bound SspCA. Each point is the mean of three independent determinations.

The effect of temperature at 25, 50, and 70 °C was determined for the free SspCA, and the whole cells surface and outer membrane displaying SspCA as shown in Figure 5. In Figure 5, we have reported only the results obtained for the outer membrane displaying SspCA because the whole cells had a behaviour very similar to that of the outer membrane fraction. On one hand, the residual activity of the membrane-bound SspCA remained almost constant at both 25 °C and 50 °C for all times considered on the *x*-axis (Figure 5); at 70 °C and after 5 h, the residual activity decreases to 70%, but remained constant at this value for all the time considered (15 h). On the other hand, the free SspCA was less stable compared to the membrane-bound SspCA. In fact, at 25 and 50 °C and after 5 h the residual enzyme activity was about the half respect to the bound enzyme (see Graph at 25 °C and 50 °C). At 70 °C, the residual activity of the free SspCA became less than 20% (Figure 5, 70 °C). Thus, by increasing the incubation time, the unbound enzymes showed a different behaviour compared with the membrane immobilised ones. In particular, the free SspCA residual activity at 70 °C became 20% after 5 h, while that of the membrane SspCA was 70% for all the time. These results demonstrated that the anchoring system considerably increased the SspCA stability. The membrane-bound enzymes continued to work for several hours at temperature considered prohibitive for free enzymes, such as 70 °C. This is an interesting aspect considering the fact that the temperature of the absorption column used for the biomimetic capture of CO_2_ typically ranges between 40 °C and 60 °C. Of course, these temperatures are critical for the bacterial cells expressing the SspCA on the cellular surface, but not for the enzyme that can continue to function even if the bacterial cells are killed by the high temperature. Therefore, the whole cells expressing the thermostable SspCA represent only a support for making more stable an enzyme itself already stable. As demonstrated for the immobilisation of the enzyme onto magnetic nanoparticles, the membrane-bound enzyme immobilised on the cell surface is a good choice for enhancing the operational stability of the enzymes.

### Long-term stability

**Figure 6.**
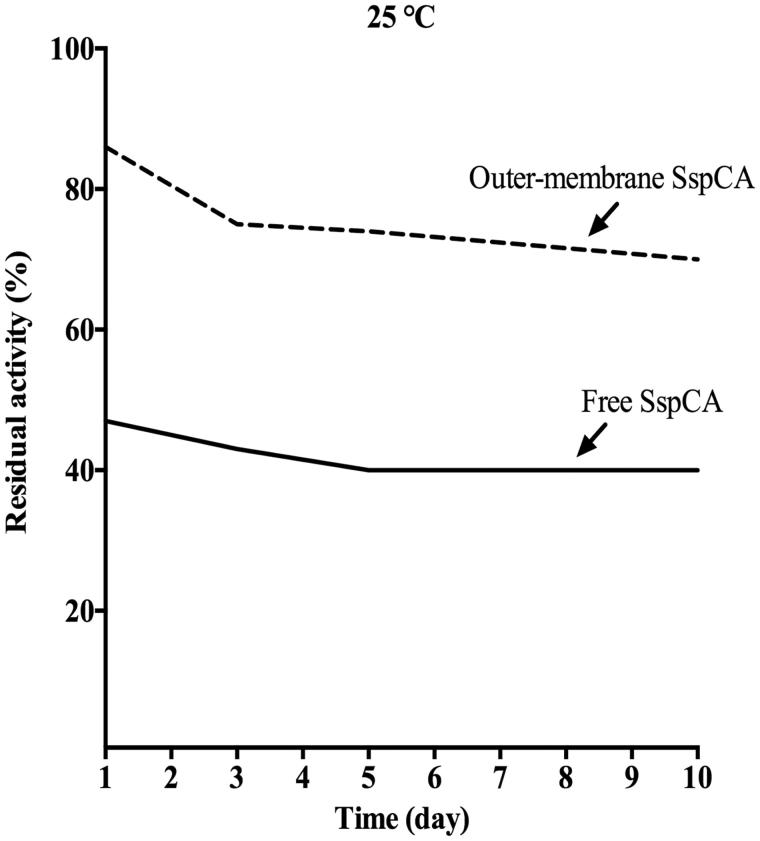
The long-term stability of free and membrane-bound SspCA. Long-term stability was performed at 25 °C measuring the residual activity of the free and membrane-bound SspCA at the days indicated on the *x*-axis. Continuous line: free SspCA; dashed line: membrane-bound SspCA. Each point is the mean of three independent determinations.

In Figure 6, the long-term stability of the free and the outer membrane-bound SspCA at 25 °C is shown. After an incubation time of 10 d, the residual activity of the free SspCA was 40% (Figure 6). Interestingly, the bound SspCA decrease its residual activity at about 70% after 10 d of storage at 25 °C (Figure 6). These results clearly demonstrated that the storage stability of the enzymes was significantly improved after anchoring the SspCA to the bacterial membrane.

## Conclusions

On one hand, the Gram-positive bacteria are microorganisms stained by the dye proposed by Gram and generally contain a thick cell wall that is very rich in cross-linked peptidoglycans, but also teichoic acids, teichuronic acid, and polysaccharides^76^. On the other hand, the Gram-negative bacteria have a thin layer of peptidoglycan and an outer membrane containing lipopolysaccharides, which lies outside of the peptidoglycan layer, and the Gram reagent does not stain them^77–79^. For obtaining the surface exposure of heterologous proteins on the outer membrane of Gram-negative bacteria, it is possible to use natural systems of the microorganism, such as OmpA, chimeric OmpA, INP, etc., which serve as a carrier of heterologous gene products to be displayed at the outer surface of Gram-negative bacteria^80^ ^,^ ^81^. INP is one of the most effective display systems for Gram-negative bacteria available. *P. syringae* has an ice nucleation protein (INP) that resides on the surface of cells. INP is anchored to the outer membrane via the glycosyl-phosphatidylinositol (GPI)-anchor sequence^80^ ^,^ ^81^. By fusing the thermostable SspCA to the C-terminus of INPN domain of *P. syringae* and expressing this construct in *E*. *coli*, the recombinant bacteria obtained a surface-localised SspCA activity. Determining the hydratase activity of the immobilised SspCA on the outer membrane, and using techniques, such as the protonography and the Western blot, we proved that INPN-SspCA could be secreted and targeted to the outer membrane. Moreover, taking into account that the membrane immobilised SspCA fusion protein was stable for 15 h at 70 °C and, for days at 25 °C, we can conclude that the INPN-SspCA anchoring system or the engineered bacteria could be considered as a good strategy to be used for the biomimetic capture of CO_2_ and other biotechnological applications in which a highly effective, thermostable catalyst such as SspCA is needed.
